# Development of 3D Models of Knits from Multi-Filament Ultra-Strong Yarns for Theoretical Modelling of Air Permeability

**DOI:** 10.3390/ma14133489

**Published:** 2021-06-23

**Authors:** Tetiana Ielina, Liudmyla Halavska, Daiva Mikucioniene, Rimvydas Milasius, Svitlana Bobrova, Oksana Dmytryk

**Affiliations:** 1 Department of Textile Technology and Design, Kyiv National University of Technologies and Design, 01001 Kyiv, Ukraine; yelina.tv@knutd.edu.ua (T.I.); galavska.ly@knutd.edu.ua (L.H.); bobrova.sy@knutd.edu.ua (S.B.); dmytryk.om@knutd.edu.ua (O.D.); 2 Department of Production Engineering, Kaunas University of Technology, 44249 Kaunas, Lithuania; daiva.mikucioniene@ktu.lt

**Keywords:** knit, plain stitch, multi-filament yarn, 3D model, stretching, air permeability

## Abstract

The work is devoted to the study of the geometric parameters of a knitted loop. It has been found that the optimal model is a loop model detailed at the yarn level, which considers the change in the cross-sectional shape and sets the properties of the porous material in accordance with the internal porosity of the yarn. A mathematical description of the coordinates of the characteristic points of the loop and an algorithm for calculating the coordinates of the control vertices of the second order spline, which determine the configuration of the yarn axes in the loop, are presented in this work. To create 3D models, Autodesk AutoCAD software and Structura 3D software, developed in the AutoLisp programming language, were used. The simulation of the air flow process was carried out in the Autodesk CFD Simulation environment. For the experimental investigation, plane knits from 44 tex × 3 linear density ultra-high molecular weight polyethylene yarns were produced, and their air permeability was tested according to Standard DSTU ISO 9237:2003. The results obtained during the laboratory experiment and simulation differed by less than 5%.

## 1. Introduction

Current approaches to the modelling of the physical properties and mechanical behaviour of knitted structures are based on the use of specialized software. To a greater or lesser extent, theoretical models consider the internal hierarchy of textile structures and provide an opportunity to choose the optimal level of modelling. They are usually based on the use of the principle of homogenization and consist of generalizations of the properties of the structural parts of the lower level in their transition to the upper one. The methods of homogenization have received the greatest development in relation to predictions of the properties of textile reinforced composite materials [1,2,3]. Studies have also shown the application of various theoretical approaches to the homogenization of the properties of textile fabrics and products. The multiscale hierarchical model for the determination of the properties of woven fabrics was proposed by Carvelli and Poggi [4]. Multiscale modelling systems that support the interaction between fine-scale and coarse-scale models make it possible to assess material properties based on the fine-scale details. Talebi et al. developed an open-source framework for multiscale modelling and the simulation of cracks in solids [5]. In order to form a theoretical background of the software, three categories of multiscale modelling techniques were investigated: hierarchical, semi-concurrent, and concurrent. However, a knitted structure cannot be considered as a continuum, but rather as a rod construction and requires other homogenization algorithms. The results of computer simulation depend on the level of geometric modelling. A representation of the geometry of the yarn (knitted into a stitch) and its structural parts, such as single-strand yarns, filaments or even fibres, is a rather difficult task. In addition, micro level modelling is associated with an increase in the computational time. When deciding on the level of detail of the model, it is important to assess the relevance of the complexity of the model geometry. On the other hand, attempts to simplify the geometry of the model and the transition to the macro level can give adequate results only if there are reliable, proven algorithms that consider their physical properties and structural parameters.

An assessment of the air permeability of textile fabrics is important in the context of ensuring the necessary functionality of filter materials, sportswear, outerwear and underwear, medical and rehabilitation products, as well as other groups of textile products. The air permeability of knitted structures depends on many factors, the most important of which are the type of raw material, the parameters of yarns, the knitting pattern, the technical parameters of the knitting process, the operating conditions, the processing methods, etc. Cotton and other natural fibres are often used for knitted clothes because of the high air and water vapor permeability. At the same time, it is strongly recommended to combine natural and synthetic fibers; this allows for better operational characteristics [6,7,8,9]. Wilbik-Hałgas et al. used a computer image analysis for an assessment of the surface porosity of knitted fabrics. According to the results of the study, air permeability is a function of the structural characteristics of knitted fabrics, such as surface porosity and thickness [10]. In their research [11], Muraliene and Mikucioniene investigated the changes in the air permeability of knitted samples with an elastomeric in-lay yarn insertion and without it in a stretched state. Špelić et al. studied the effects of walking on the thermal properties of clothing and subjective comfort [12]. Thermo-mechanical technological processes that textile materials undergo in the manufacturing process may affect their geometrical and physical properties [13]. The air permeability of textile materials used for clothing greatly influences a person’s sense of comfort. There are theoretical models of the air permeability of knitted fabrics presented in the literature [14,15,16,17]. Ogulata and Mavruz [16] presented the relationship between the porosity of knitwear and its air permeability and proposed a method for its theoretical determination. The results of the use of this technique in the computer modelling of air permeability are presented in [17]. Air permeability is an important characteristic and is unique to materials which exhibit porosity. Usually, there are two types of pores in the structure of textile fabrics: inter-structural (inter-yarns) spaces and air channels in yarns. Xiao et al. [18] considered the pores of textile fabrics as tubes with variable cross sections and, considering the curvature of the pore walls, presented expressions describing the air flow. Another expression connecting the drop of air pressure with the dynamic viscosity of air, the air flow rate, as well as the length and hydraulic diameter of the pore, was given in the work of Kulichenko [19]. In the presence of through-structural pores, the air permeability of the textile material is mostly determined by the through-structural porosity. Moreover, the size and configuration of the pores are also important. Thus, for the same value of the through-structural porosity, fabric made from the thin yarns will have a lower air permeability than a fabric made from the thick yarns [19]. With an increase in the size of through-structural pores, the significance of the fibrous composition as a factor in the air permeability of the textile fabric decreases. However, the hairiness and roughness of the yarn creates aerodynamic friction on the surface of the yarn and determines an increase in the resistance to air flow. Therefore, the hairiness and roughness of the yarn surface reduces the flow rate and, accordingly, reduces the air permeability. Moreover, the orientation and method of fixing the fibers and individual sections of the yarn in the fabric structure are also important [14].

Due to an increased interest in the 3D modeling of knitted structures, many papers have been dedicated to the development of a geometric description of a knitted stitch. Different approaches have been suggested by Kyosev et al. [20], Trujevcev [21], Kaldor et al. [22], Wadekar et al. [22], and others. Topological models for weft knitted structure descriptions are proposed in [23,24,25]. Puszkarz and Krucinska [26] investigated the air permeability of two-layer knitted fabrics experimentally and by means of computer modeling. The authors considered the compliance of the results. A comparison of the experimental results and those obtained during the simulation of the process of air flow through models of the knitted structure, detailed at the level of single yarns and at the level of filaments, confirmed that the data obtained by using models, detailed at the level of filaments, were closer to the experimental results. The study of knitted fabric thermal insulation [27], realized with mono-fiber and multi-fiber 3D models, showed that both measurements and simulations yielded comparable results. 

Later scientific publications have enhanced the theoretical approaches to the development of materials-by-design. Ultra-high molecular weight polyethylene (UHMWPE) threads can be used for the production of protective clothes. An optimization of the design process of such items can be reached by studying their physical and mechanical behavior and improving the means of creating their three-dimensional models. It is necessary to consider the possibility of modeling deformations in knitwear samples yielding to changing physical properties. In this study we focus on the peculiarities of unidirectional stretching and an air permeability simulation of plain knits made from UHMWPE threads.

## 2. Materials and Methods

Computational fluid dynamics (CFD) methods are based on the continuity equation, which in the case of a liquid or gas is reduced to the Navier-Stokes’ equations and the equations of conservation. Modern software systems are equipped with a mathematical apparatus to solve a system of differential equations that describe the task and consider the geometry of the three-dimensional model and the user-specified boundary conditions. In order to simplify the work with porous materials, popular CFD programs provide the ability for the permeability, or the porosity of materials to be considered by setting one of the available indicators, depending on the available set of initial data. For example, the Autodesk CFD Simulation software [28] used for modelling a porous object provides the option to choose such a type of material as the so-called distributed resistance to flow. Such materials can be characterized by an additional pressure drop after flowing through the material. A description of the characteristics in the settings of the material can be achieved by one of the possible ways:

- through the pressure drop coefficient, where the overpressure gradient can be determined using the Expression (1):
  (1) $$\frac{\partial p}{\partial X_{i}}=K_{i}\frac{\rho V_{i}^{2}}{2}$$
  where *i* is the direction of the global coordinate. Index *K* may be found by determining the pressure drop against flow.
- through the friction index [14]. In such case, the gradient of additional pressure can be expressed by the Equation (2):
  (2) $$\frac{\partial p}{\partial X_{i}}=\frac{f}{D_{h}}\frac{\rho V_{i}^{2}}{2}$$
  where *f* is the coefficient of friction; *D_h_* is the hydraulic diameter in mm.
- through the Darcy ratio (3):
  (3) $$\frac{\partial p}{\partial X_{i}}=C\mu Vi$$
  where *C* is the filling factor which is the inverse value to the permeability; *μ* is the viscosity of the flow [14].

In [19], a dynamic model of the isothermal filtration of low humidity air through multilayer porous structures was proposed. The dependence of the conductivity on the rate of air filtration in packets of barrier clothing as quasi-linear generalizations of Darcy’s law was established. According to this model, the air velocity in the pore ***V*** (m/s) can be calculated using the Expression (4):

(4) $$V=\frac{\Delta P}{80\mu }\cdot \left(\frac{d_{h}^{2}}{L}\right)$$

where Δ*P* is the differential pressure with the laminar flow of liquid or gas; *μ* is the dynamic viscosity of liquid or gas in Pa·s; *d_h_* is the hydraulic diameter of the pore in mm; *L* is the thickness of the porous material in mm.

The hydraulic diameter of the pore is equal to:

(5) $$d_{h}=4\cdot \left(\frac{S}{P}\right)$$

where *S* is the area of the pore in mm^2^; *P* is perimeter of the pore in mm.

**Figure 1:**
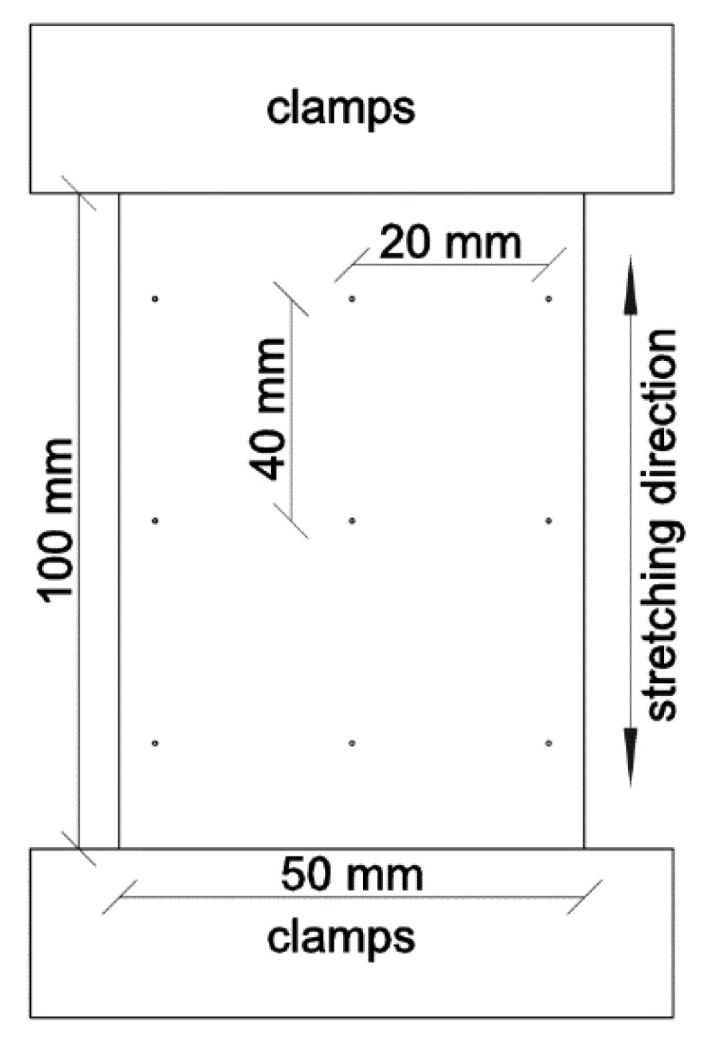
Schematic of the stretching of a knitted sample.

For experimental investigations, 3 samples were knitted on the flat weft-knitting machine of gauge 8E from DOYENTRONTEX with 44 tex ×3 linear density ultra-high molecular weight polyethylene yarns (UHMWPE) in the single jersey pattern. To stretch knitted specimens in a uniaxial direction along wales and courses, the tensile-testing machine Kao Tiech KTO-7010AZ (Beijing TIME High Technology Ltd., Beijing, China) was used. Knitted samples of 200 mm × 50 mm size were prepared and a size of 100 mm was marked for fixation in the clamps of the tensile tester. On each sample the control points were marked as shown in Figure 1.

**Figure 2:**
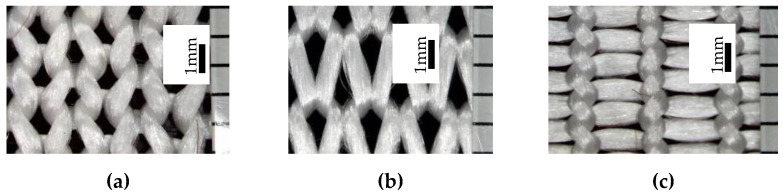
Images of knitted samples: (**a**) in a relaxed state; (**b**) after being stretched along wales; (**c**) after being stretched along courses.

Macrophotographs of the knitted structure were made before and after the stretching forces were applied by means of the digital microscope Microsafe ShinyVision MM-2288-5X-S. (Taiwan) After the stretching distance between the control points changed, however, the number of stitches did not. The course and wale spacing was assessed by dividing the average distance between the control points by the numbers of stitches in the corresponding direction. Structural parameters were determined for the knitted fabrics in a relaxed stable state (Figure 2a) and after maximal unidirectional stretching along wales (Figure 2b) and courses (Figure 2c). Images of these structures are presented in Figure 2.

The experimentally determined structural parameters of the knitted specimens before stretching and after maximal stretching in wale and course directions are presented in Table 1.

Air permeability is characterized by the amount of air (dm^3^) that passes through 1 m^2^ of textile fabric in 1 second at a certain pressure difference on both sides of the fabric. The air permeability of knitted fabrics was determined in accordance with DSTU ISO 9237: 2003. Ten experimental tests were performed for each sample variant. All measurements were carried out in a standard atmosphere according to Standard EN ISO 139:2005 (20 °C ± 2 °C temperature and 65% ± 4% humidity).

## 3. Results and Discussions

### 3.1. Modelling of the Knitted Loop

**Figure 3:**
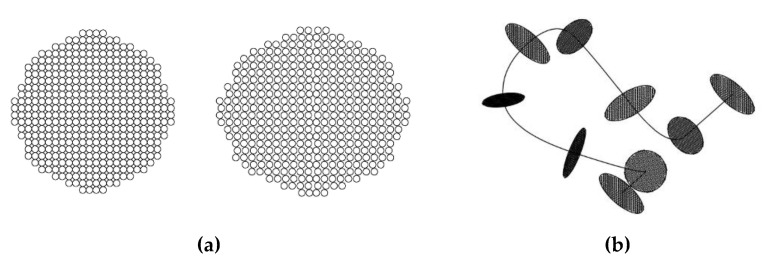
(**a**) Principal arrangement of the cross-sections of individual filaments in the structure of the yarn and (**b**) in the yarn bent into the loop.

The geometrical description of a yarn bent into a knitted loop includes the option to describe the axial line of the yarn and the pattern of the changes in the shape and size of the yarn along its axial line. In this research, the model described in [29,30] was used for the determination of the axial line geometry, as the yarn used for both the experimental knitted fabrics and the theoretical modelling was multifilament and consisted of 420 filaments, each of which had a diameter of 0.025 mm. To construct the volume of the yarn, a monofilament and multifilament model was considered as an option for use. The shape and size of the sections (the boundaries of the dense bundle of filaments) changes along the axial line of the yarn. Microlevel models are built in AutoCAD using Structura-3D software in addition. The selected method of (a) the arrangement of the cross-sections of the individual filaments in the structure of the yarn and (b) the alternation of circular and elliptical sections of the yarn along its axial line is shown in Figure 3.

**Figure 4:**
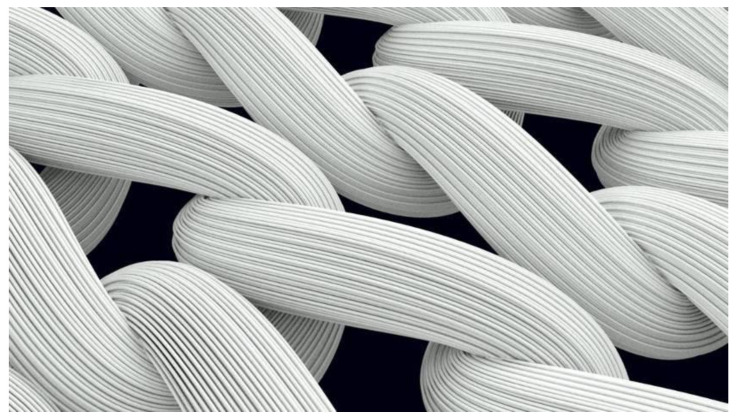
Visualization of the knitted fabric structure with detailing at the level of individual filaments, built in the Autodesk AutoCAD software environment.

A visualization of a structural fragment of the knitted sample, built considering the geometry of the individual filaments in the yarn, is presented in Figure 4. However, the disadvantages of this model are a significant mutual penetration (cross-section) of the volumes that represent individual filaments in the model, and high demand for RAM.

**Figure 5:**
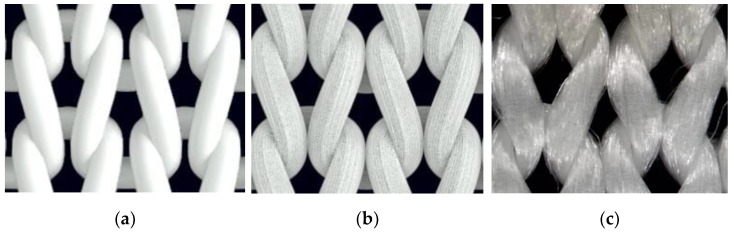
(**a**) Model of a knitted sample in a free state detailed at the level of yarn; (**b**) individual filaments; (**c**) image of the knitted sample in a free state.

A comparison of the models of different detailing levels with a photographic image of the corresponding knitted sample (made by using digital microscope Microsafe ShinyVision MM-2288-5X-S (Taiwan)) is shown in Figure 5.

**Figure 6:**
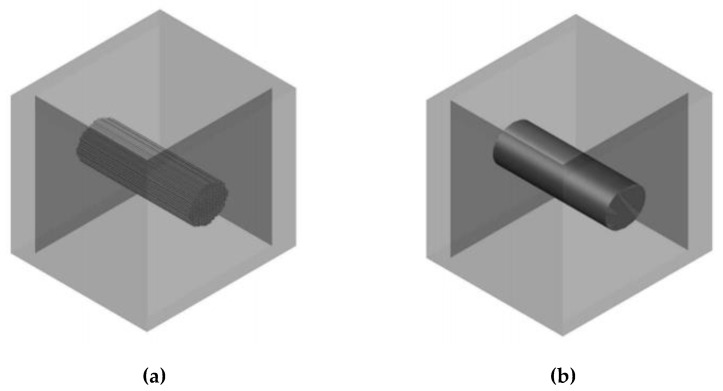
Models of a straight yarn segment made with filaments (**a**) and without filaments (**b**).

**Figure 7:**
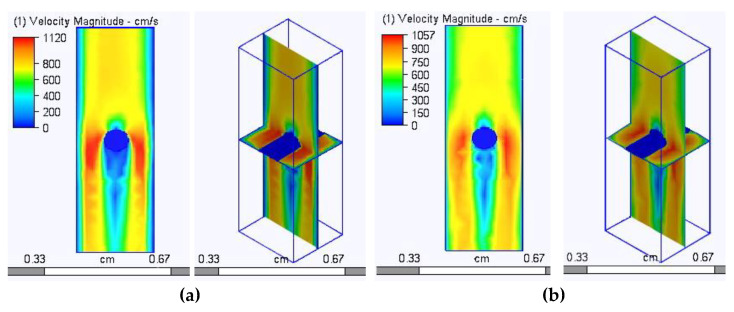
Graphical results of the analysis of the air flow through a tube with a linear yarn fragment made as multi-fiber (**a**) and mono-fiber (**b**).

As has been mentioned, the presence of sufficiently large through-structural (inter-yarn) pores in the case of a dense arrangement of filaments in the structure of the yarn means that air passes only through these pores, since the distance between the individual filaments does not exceed the thickness of the boundary layer. In addition, according to Bernoulli’s law, when passing through narrow sections, the air flow velocity increases, while in the zone of increased velocity, the pressure decreases, which leads to the suction of an additional volume of air adjacent to these flow tubes. To illustrate this phenomenon, we developed in the Autodesk AutoCAD environment the model of a straight filament fragment detailed at the level of filaments (Figure 5a) and the monofilament model of a yarn fragment with an equivalent volume (Figure 5b). These models were placed in tube models of identical geometric parameters and imported into the Autodesk Simulation CFD environment, where the same boundary conditions were set at a pressure drop of 49 Pa. Graphical results of the analysis are presented in Figure 6 and Figure 7.

In Figure 7, where the zones of the highest air flow velocity are shown in red and the lowest in blue, the program algorithms considered the set of filaments (bundle) impenetrable (Figure 7a) and the air bent around the yarn fragment, as it is shown in Figure 7b.

The obtained data from the calculations show that the filtration rate is not higher, but lower than in the case of an impermeable and smooth monofilament yarn model. This can be explained by the greater surface roughness of the multifilament model because the longitudinal protrusions on the surface form ribbing, with a height equal to the radius of an individual filament (in our case, 0.0125 mm).

In order to validate the geometric model of a stretched plain knitted structure, both laboratory and simulation methods of air permeability assessment were completed. To simulate the process of air flow through the structure of knitted fabrics, models with geometry detailed at the yarn level, with an adjustment of the flow resistance coefficient, were selected. As the initial data for the algorithm of selected modeling [29], the following parameters were provided: wale spacing ***w***, course spacing ***c***, thickness of the knitted fabric ***M***, yarn diameter ***D***, and the tangent angle at the interlacing point ***γ***, as well as the angle of inclination of the loop legs ***α*** (as shown in Figure 9b). These initial data can be obtained experimentally or calculated according to well-known methods, for example, those presented in [21]. For the model of the knitted structure, maximally stretched along wales, ***w = w_max_*** and ***c = c_min_***, while for the model of knitted structure, maximally stretched along courses, ***w = w_min_*** and ***c = c_max_***. The effective radius ***R_0_*** of a multifilament yarn [31] can be determined by the formula (6):

(6) $$R_{0}=\frac{d}{2}\sqrt{\frac{N}{\varphi^{\prime }}}$$

where *d* is the diameter of the elementary filament in mm; *N* is the number of filaments in a yarn; *φ* is the coefficient of the packing density.

In the case of the ultimate packing density, *φ* = 0.92. Then, for our multifilament yarn *R_0_*:

(7) $$R_{0}=\frac{0.025}{2}\sqrt{\frac{420}{0.92}}=0.267$$

It can be assumed that the diameter of the yarn in the compressed state is *D_0_* = 0.534 mm.

**Figure 8:**
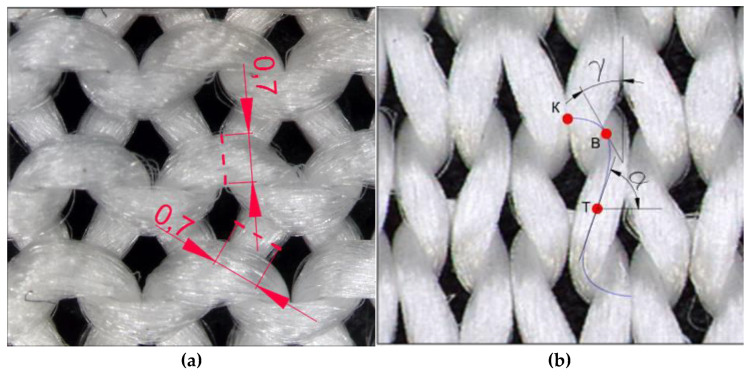
Layout of the elliptical section (**a**); and characteristic points (**b**).

It is known that in the process of stretching the knitted single jersey fabric along the wales, the wale spacing ***w*** takes the value ***w_min_*** when the yarn is redistributed and pulled from the loops’ heads and feet into the loop legs. In this case *w_min_ =* 4*·D_0_*, id est, the minimum wale spacing is equal to four minimum yarn diameters (because the yarn is in a compressed state). If we accept the assumption of a circular cross-section of the yarn, then the value of the minimum wale spacing, considering Expression (6), will be equal to 2.14 mm, which significantly exceeds the experimentally obtained value for the stretched knitted fabric, which is 1.58 mm (see in Table 1). However, if the bundle of filaments has an elliptical cross-section, and the position of the major and minor axes of the ellipse changes along the axial line of the yarn as shown in Figure 8b, the ratio of the values of the major and minor semiaxes of the ellipse can be selected, at which the area of the ellipse is equal to the area of the circle of the radius determined by Equation (6), and their projection onto the plane of the fabric is equal to the width of the projection of the yarn onto the plane of the fabric, determined experimentally, as shown in Figure 8a.

**Figure 9:**
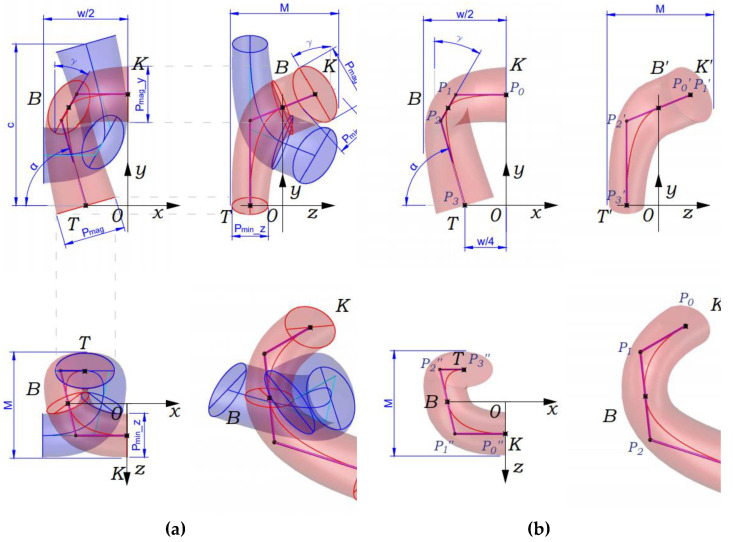
Characteristic points K, B, T (**a**) and the control points P0, P1, P2, P3 (**b**) of the spline curve, which describes a quarter of the axial line of the yarn in the knitted loop of single jersey structure.

As it is presented in [30], when simulating in a 3D environment, a space curve representing the axial line of the yarn in the model must pass through the characteristic points that lie directly on the axial line of the yarn and are located so that in 3D space their position can be determined using traditional ideas about the shape of the loop. In Figure 8a, the schematical view of the area of the interlacing of the yarns in the loop of the single jersey knitted fabric is presented. Characteristic points K, B, T, which belong to the axial line of the yarn, and control points P_0_, P_1_, P_2_, P_3_, which lie at the intersection of tangents to the axial line drawn at points K, B and T, are shown accordingly in Figure 9a,b. To describe the axes of the ellipse, the following notation is used: P_max_ is the large, and P_min_ is the minor axis of the ellipse. The axial line equation is built on the basis of the coordinates of the control points, and those, in turn, can be calculated based on traditional ideas about the geometry of the knitted loop, taking into account certain assumptions.

**Figure 10:**
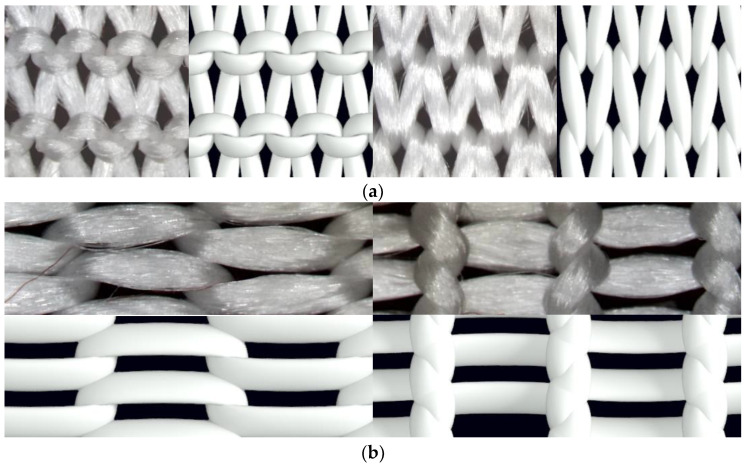
Meso-model and image of the single jersey knitted fabric made from high molecular weight polyethylene yarns at maximum stretching along wales (**a**) and along courses (**b**).

In the coordinate system, situated as shown in Figure 10, the coordinates of points *K* (X_k_, Y_k_, Z_k_), *A* (X_a_, Y_a_, Z_a_), *B* (X_b_, Y_b_, Z_b_), *C* (X_c_, Y_c_, Z_c_), *T* (X_t_, Y_t_, Z_t_) can be calculated taking into account the accepted assumptions about the symmetric shape of the loop and the known characteristics of the structure: *w*, *c*, *P_max_*, *P_min_*, *M*. Mathematical expressions for determining the coordinates of the characteristic points of the loop in the selected coordinate system are presented in Table 2.

The direction of the tangents to the points *K*, *B* and *T* in the projection onto the XOY plane can be determined as follows. The tangent at point *K* (segment P_0_P_1_) is parallel to the OX axis and the XOY plane (plane of the fabric). In the projection onto the YOZ plane, the segment P_0_P_1_ is expressed to a point that coincides with the point *K*. The tangent at point *T* (segment P_2_P_3_) is located at an angle (180 − α) to the X-axis. It is also parallel to the XOY plane but located on the opposite side of the XOY than the segment P_0_P_1_. This is clearly seen in the projection onto the XOZ plane (Figure 10b). The segment P_1_P_2_ intersects with the plane of the fabric (XOY) at point *B*. Point P_1_ belongs to the tangent line drawn through the point *K*, and point P_2_ belongs to the tangent line drawn through the point *T*. In accordance with the previously accepted assumptions, the projection of the tangent at point *B* onto the plane of the fabric is located at an angle *γ* to the OY axis.

The presented algorithm of the calculation allows the coordinates of the characteristic points of the loop and the direction of the tangents at these points to be determined. It allows the creation of a *B*-spline, which reproduces the axial line of the yarn in 3D modelling systems of the knitted structure. Since in systems of finite element modelling mechanical properties are assigned to individual design objects, in meso-models used for the modelling of deformations, it is advisable to use the cross-section of the yarn in a compressed state in all sections of the loop.

The three-dimensional model and the images of the sample of single jersey knitted fabric made of high molecular weight polyethylene yarns at maximum stretching along the wales are presented in Figure 10a, and along courses in Figure 10b. The yarn cross-section of the model is shown as an ellipse.

### 3.2. Determination of Air Permeability and Its Simulation

In order to simulate a laboratory experiment in the Autodesk Simulation CFD software environment for a three-dimensional model, the boundary conditions were set as following: a pressure drop corresponded to 5 mm water column height or 49 Pa; the environmental temperature was set at 20 °C. 

The internal porosity of the yarn was set at 8% for all specimen models. The results of air permeability obtained experimentally and during simulation using the Autodesk CFD Simulation software are presented in Table 3.

**Figure 11:**
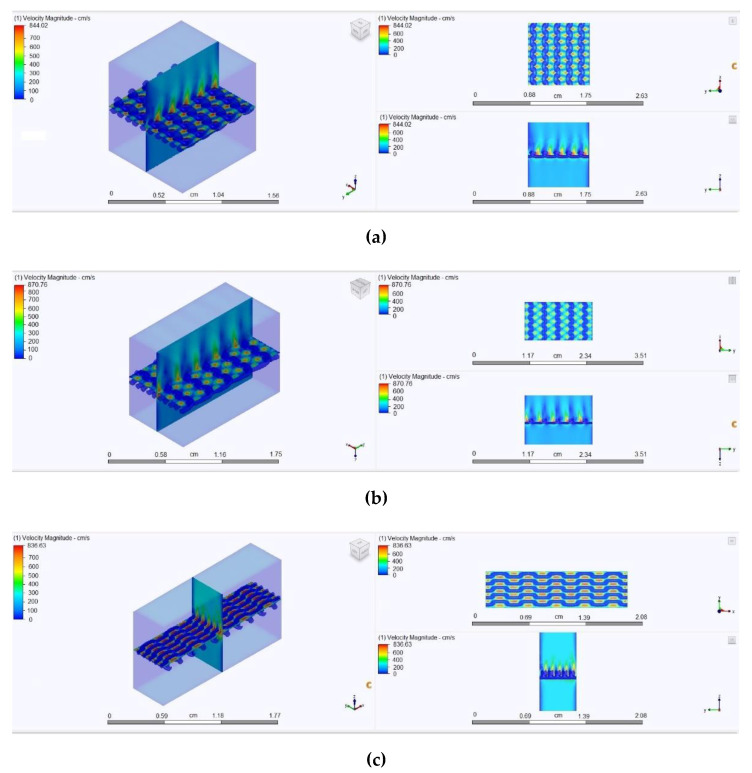
Illustration of the process of air passing through the knitted fabric under the condition of a pressure drop of 49 Pa: (**a**) model of the knitted fabric in a free state, (**b**) model of the knitted fabric in the state of uniaxial stretching along the wales, (**c**) model of the knitted fabric in the state of uniaxial stretching along the courses.

The images obtained during the analysis of the simulation results are presented in Figure 11. The images present the distribution of zones with different air velocities. In accordance with the theory laid down in the algorithms of the program, the highest air velocity was in the narrowest places (shown in red in the diagrams). The flow rate decreased at the pore walls, which also corresponded to the positions of hydro and aerodynamics, which explains the decrease in the flow rate when approaching the tube (pore) walls due to the viscosity of the liquid or gas and friction forces.

A comparison of the air permeability results obtained experimentally and after virtual simulation confirmed good accuracy. However, it should be noted that in real textile materials, the air flow can change the geometry of the holes depending on the pressure drop and the strength of the fastening of individual sections of the yarn in the knitted fabric. So far, it is difficult to take this into account in the available geometric models of knitted structures and the physical models of processes. Further research in this direction will improve the accuracy of the results obtained during the simulation.

## 4. Conclusions

Current trends in the use of digital tools for the modelling of physical processes occurring in knitted structures, particularly air permeability, were examined in this research. The features of constructing the model of the loop knitted in single jersey pattern, detailed at the level of yarns and individual filaments, were considered in this work. It was proposed that the air channels formed between the individual filaments in the yarn with special characteristics of porous materials were considered. The experimental results of the air permeability of single jersey knitted fabric from 44 tex ×3 linear density ultra-high molecular weight polyethylene yarns confirmed good accuracy of the model implemented using the Autodesk CFD Simulation software. The results obtained allow the proposed yarn-level geometric model to be used in assessments of the air permeability of plain knitted fabrics from synthetic hydrophobic yarns. Further development of the model could be performed by studying the hydraulic diameter changes of the pores under air or liquid flow in order to enhance its applicability for hydrophilic yarns. Also, experimental verification of the presented mathematical model will be done during the further investigations.

## Figures and Tables

**Table 1:** Structural parameters.

Stretching Direction	Wale Spacing *w*, mm	Course Spacing *c*, mm	Yarn Diameter *D*, mm	Fabric Thickness *M*, mm	Loop Length *l*, mm
Before stretching	1.92 ± 0.1	1.85 ± 0.1	0.7 ± 0.05	0.85 ± 0.05	7.95 ± 0.4
Stretched along wales	1.58 ± 0.1	2.78 ± 0.15		0.75 ± 0.05	
Stretched along courses	3.46 ± 0.2	0.85 ± 0.05		0.82 ± 0.05	

**Table 2:** Mathematical expressions for determining the coordinates of characteristic points in coordinate system of the loop.

Point	Abscissa	Ordinate	Applicata
***K***	$X_{k}=0$	$Y_{k}=\frac{c+P_{mag}}{2}$	$Z_{k}=\frac{M-P_{\min}{\_}_{z}}{2}$
***B***	$X_{b}=\frac{w}{4}+\frac{P_{\min}}{2}\cos\gamma$	$Y_{b}=\frac{c}{2}+\frac{D_{y}}{2}\sin\gamma$	$Z_{b}=0$
***T***	$X_{t}=\frac{A}{4}$	$Y_{t}=0$	$Z_{t}=-\frac{M-P_{\min}}{2}$

**Table 3:** Air permeability of the single jersey knitted fabric.

Sample No	Experimental Value of Air Permeability, dm^3^/(m^2^s)	Simulated Value of Air Permeability, dm^3^/(m^2^s)	Discrepancy of the Simulated Value, %
1	1617	1687	0.04
2	2353	2301	−0.02
3	1646	1645	0.00
